# Comparing end-user diagnostic outputs from a commercial tNGS pipeline for *Mycobacterium tuberculosis* drug resistance detection

**DOI:** 10.5588/ijtldopen.25.0245

**Published:** 2025-11-12

**Authors:** M. Seifert, R.E. Colman, S. Laurent, A. De la Rossa, S. Uplekar, C. Rodrigues, N. Tukvadze, S.V. Omar, A. Suresh, T.C. Rodwell

**Affiliations:** 1 FIND, Geneva, Switzerland;; 2 Division of Pulmonary, Critical Care, Sleep Medicine, and Physiology, University of California San Diego, La Jolla, CA, USA;; 3 Hinduja Hospital and Medical Research Centre, Tuberculosis, Mumbai, India;; 4 National Center for Tuberculosis and Lung Diseases, Tuberculosis, Tbilisi, Georgia;; 5 Swiss Tropical and Public Health Institute, Allschwil, Switzerland;; 6 University of Basel, Basel, Switzerland;; 7 Centre for Tuberculosis, National and WHO Supranational TB Reference Laboratory, National Institute for Communicable Diseases, A Division of the National Health Laboratory Service, Johannesburg, South Africa.

**Keywords:** tuberculosis, diagnosis, targeted next-generation sequencing, pipeline updates, drug resistance

## Abstract

**BACKGROUND:**

Targeted next-generation sequencing has emerged as a rapid solution for diagnosing drug-resistant TB (DR-TB) directly from clinical specimens. Updating the bioinformatics software component can lead to rapid improvements in diagnostic performance. We compared the diagnostic performance of an updated bioinformatic pipeline output to the original pipeline output for the Oxford Nanopore Technology (ONT) TB Drug Resistance Test.

**METHODS:**

A total of 721 sediment samples were evaluated for 13 anti-TB drugs using phenotypic drug susceptibility testing and whole genome sequencing. Sequencing data outputs previously analysed using the original pipeline were re-analysed using an updated pipeline and compared.

**RESULTS:**

There were no significant differences in successful sequencing results, and direct comparison of DR-TB call agreement was substantial (κ > 0.7) between the original and updated pipeline outputs. Diagnostic accuracy relative to the composite reference standard was compared, and significant (*P* value < 0.05) increases in sensitivity and diagnostic yield, using the updated pipeline, were identified for streptomycin, pyrazinamide, bedaquiline, and clofazimine.

**CONCLUSION:**

Comparison of the updated pipeline to the original pipeline revealed significant improvements in diagnostic performance, demonstrating that bioinformatic enhancements alone – without wet-lab modifications – can substantially boost sensitivity and diagnostic yield for DR-TB. These findings underscore the critical role of continuous pipeline optimisation in the evolving resistance landscape to enhance real-time clinical decision-making.

In recent years, targeted next-generation sequencing (tNGS) has emerged as a promising approach for diagnosing drug-resistant TB (DR-TB), offering the ability to detect comprehensive resistance phenotypes associated with mutations in the genome of the causative organism, *Mycobacterium tuberculosis*, directly from clinical specimens at relatively low cost.^1,2^ Compared to more traditional whole genome sequencing (WGS) methods, tNGS allows for higher throughput and reduced computational burden by enriching for gene targets or sequences of interest.^3–5^ Bioinformatic pipelines, which are integral to the overall performance of tNGS, process raw sequencing data into clinically interpretable results by mapping reads to a reference genome, identifying variants, ensuring the quality of the variant calls, and comparing variants to mutations known to be associated with phenotypic drug resistance.^6,7^ Unlike traditional phenotypic drug susceptibility testing (pDST) or rapid molecular assays, tNGS workflows have the potential to be continuously improved through standalone bioinformatic updates as new mutations associated with resistance are identified, without altering wet-lab components and requiring full assay revalidation if the updated mutations are contained in the target regions already included in the assay. This capability enables tNGS bioinformatics pipeline analytics to evolve in real time, allowing for rapid updates that adapt to a dynamic knowledge base of resistance-associated mutations, ensuring both diagnostic accuracy and clinical utility. This flexibility is particularly relevant for DR-TB, where emerging mutations and resistance to newly introduced drugs significantly influence treatment decisions.^8–11^

In this study, we analysed previously published raw sequence data using an updated pipeline for the Oxford Nanopore Diagnostics (ONT) TB Drug Resistance Test and compared results to the original pipeline output using a composite reference of pDST and WGS (2023 WHO mutation catalogue).^12,13^ Performance metrics, including overall sequencing success, pairwise resistance call agreement, diagnostic accuracy, and an overall diagnostic yield for drug resistance detection, for the updated pipeline were compared to the original pipeline for 13 anti-TB drugs.

## METHODS

Clinical and sequence data used for this analysis were collected as part of a prospective, cross-sectional, multicentre clinical diagnostic accuracy study, the Seq&Treat trial (clinicaltrials.gov NCT04239326), which has been previously described.^13^ In brief, individuals aged 18 years and older with pulmonary TB confirmed by GeneXpert MTB/RIF or Ultra, who were at risk for or had been diagnosed with rifampicin-resistant TB and had not initiated treatment for their current diagnosis during the 7 days prior to evaluation were eligible to enrol. Individuals from three sites, the Hinduja Hospital and Medical Research Centre in Mumbai, India, the National Center for Tuberculosis and Lung Diseases in Tbilisi, Georgia, and the National Institute for Communicable Diseases in Johannesburg, South Africa, and their peripheral treatment centres were invited to participate in the study.

### Sample collection and processing

As previously described, consented participants were asked to provide sputum, either during a single collection at enrolment or combined collections at enrolment and the following day.^13^ Samples were homogenised, decontaminated, re-suspended, and then evaluated using multiple standard-of-care diagnostic methods.^13^ pDST was performed on all culture-positive samples at WHO-endorsed critical concentrations using Mycobacteria Growth Indicator Tube for 13 drugs: rifampicin (RIF), isoniazid (INH), streptomycin (SM), ethambutol (EMB), pyrazinamide (PZA), levofloxacin (LFX), moxifloxacin (MFX), kanamycin (KAN), amikacin (AMK), capreomycin (CAP), bedaquiline (BDQ), linezolid (LZD), and clofazimine (CFZ) at previously described concentrations.^13^ DNA was extracted from cultured isolates and sequenced using the Illumina MiSeq platform; raw sequencing data were processed with a standardised bioinformatics and variant detection pipeline as described previously.^13^ Genotypic DST determination was based on detection of mutations or variants identified as ‘associated with resistance’ or ‘associated with resistance–interim’ in the updated 2023 WHO mutation catalogue.^12^

### ONT workflow

The ONT tNGS workflow was performed as described previously.^13^ Briefly, 700 µL of frozen sediment underwent DNA extraction, target application, and library preparation. Pooled samples were then sequenced using MinION Mk1B, and data were processed through the ONT ‘original’ pipeline codebase with reports generated using ONT software version ‘wf-tb-amr-3993f97d’. For the ‘updated’ ONT output, FastQ files were re-analysed using cloud implementation of the pipeline codebase ONT software version ‘wf-tb-amr-v2.0.0-alpha3’. While specific changes to the pipeline codebase are proprietary, the manufacturer updated the solution software for variant calling with the goal of detecting mutations associated with resistance to critical new and repurposed drugs BDQ and CFZ, and improving sensitivity to PZA.^14^ According to the manufacturer, the software was updated based on the mutations listed in the second edition of the WHO Mutation Catalogue, and the updates include refined thresholds for control validation, variant classification (including nucleotide insertions and deletions), and summary reporting.^12^ End-user results were generated as CSV files and collated for statistical analysis. Sequence data can be found on the NCBI Sequence Read Archive and European Nucleotide Archive under the accession number PRJNA1160005.

### Data management and analysis

For both the original and updated pipelines, sequencing results were interpreted as ‘resistance detected’ (R), ‘no resistance detected’ (S), or ‘undetermined’ (U) when the sample had insufficient coverage to determine resistance status. Sequencing was considered a failure when only U calls and/or no amplicon targets were reported for all targets assessed. If an R or S call was reported for at least one drug, sequencing was considered a partial sequencing success. Samples resulting in an R or S call for all drugs were considered complete sequencing success. Composite diagnostic reference results were determined using both pDST and genotypic DST (WGS). If resistance was detected by pDST and/or a resistance-associated mutation from 2023 WHO mutation catalogue was detected using WGS, the sample was classified as resistant.^12^ If pDST and WGS both indicated drug susceptibility, then the sample was classified as susceptible. If a mutation with a U call was reported for WGS, only the pDST result was used to determine the composite diagnostic reference.

Individual drug resistance agreement, as determined by both original and updated pipelines, was compared using Cohen’s kappa. An exact test of symmetry was used to evaluate pairwise discordance. Differences in the proportion of resistance calls (number of pipeline R calls out of all 721 samples evaluated) between the two pipelines were compared using a two-sample *Z* test. Drug-specific resistance calls as determined by pDST, WGS, and composite reference were presented as percentages. Diagnostic accuracy estimates were determined by calculating drug-specific sensitivity (true positive divided by the sum of true positive and false negative) and specificity (true negative divided by the sum of true negative and false positive) with 95% confidence intervals (CIs) using the Wilson score method and compared using a two-sample *Z* test. If the pipeline did not produce an R or S call, the sample was excluded from drug-specific sensitivity or specificity calculations. Diagnostic yield was assessed by comparing the number of correctly classified resistant calls (number of pipeline R calls) to the total number of samples classified as resistant by the composite reference standard for each drug. Differences in diagnostic yield between the two pipelines were compared using a two-sample *Z* test.

### Ethical statement

All study activities were approved by the Institutional Ethics Committees of each study site as well as the WHO Research Ethics Review Committee.

## RESULTS

Of a total of 832 clinical samples initially evaluated, 721 produced R or S WGS and pDST reference results for all drugs assessed and were analysed using both original and updated pipelines (Figure). Original and updated pipelines produced drug-specific R, S, or U results, and all 721 results were used for sequencing success analysis, drug resistance call agreement, and resistance yield detection. However, only samples where the pipeline produced an R or S result were used for diagnostic accuracy comparison. The median age of participants was 36 years (interquartile range 26–49), and 487 (68%) were male.

**Figure.**
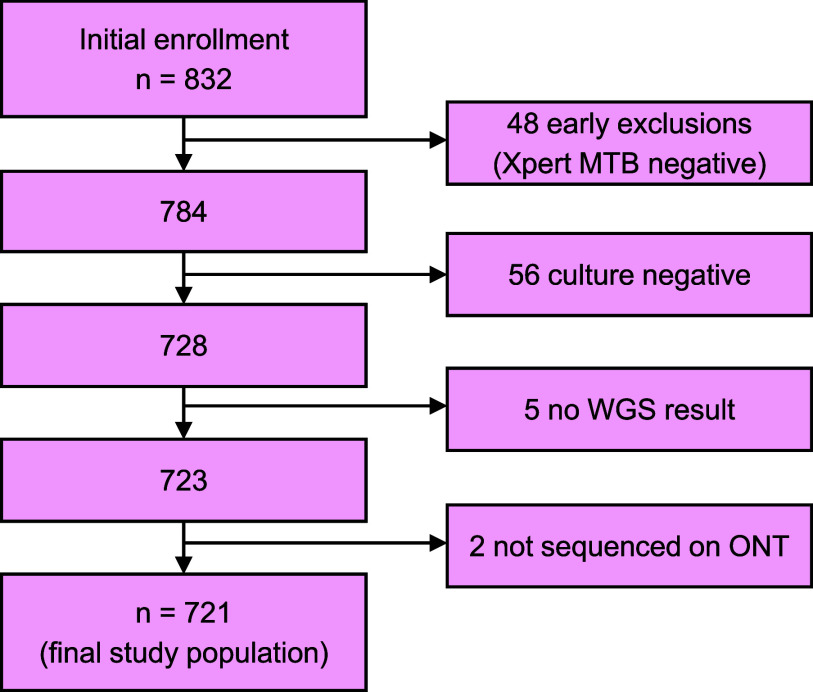
STARD flow diagram of the study. Of the 832 participants initially enrolled, 48 were excluded early due to a negative GeneXpert MTB result for the study sputum sample, an additional 61 participants were excluded due to lack of reference results (56 produced negative culture results and did not undergo drug susceptibility testing, and 5 were excluded due to lack of WGS results), and 2 participants were excluded as their samples were not sequenced using ONT, resulting in a total of 721 samples. ONT, Oxford Nanopore Technology; WGS, whole genome sequencing.

### Sequencing success

While there was no statistically significant difference (Table 1) in sequencing success when comparing the updated pipeline to the original pipeline when assessing table symmetry (Bowker’s *P* value = 0.130) or marginal homogeneity (Stuart–Maxwell *P* value = 0.127), results did vary between the original and updated pipelines. Seven samples (1.0%) failed to generate drug resistance interpretations with the original pipeline but had partial success (some drugs interpreted as R/S) with the updated pipeline. Conversely, 11 samples (1.5%) achieved partial success with the original pipeline but failed to generate any results with the updated pipeline. Additionally, 10 samples (1.4%) showed partial success with the original pipeline and complete sequencing success with the updated pipeline, whereas only 3 samples (0.4%) transitioned from complete sequencing success in the original pipeline to partial success using the updated pipeline. Notably, only one sample (0.1%) achieved complete sequencing success with the original pipeline but failed entirely with the updated pipeline.

**Table 1.** Overall sequencing success comparison between the original and updated pipelines.

Original pipeline	Updated pipeline			
	Sequencing success	Partial sequencing success	Sequencing failure	Total
Sequencing success	577	3	1	581
Partial sequencing success	10	82	11	103
Sequencing failure	0	7	30	37
Total	587	92	42	721

### Drug resistance call agreement

The proportion of samples identified as drug-resistant by the original and updated pipelines is compared in Table 2. Resistance calls for individual drugs ranged from 0% for CFZ to 71% for RIF for the original pipeline and 2% for LZD to 71% for RIF for the updated pipeline. The proportion of resistance calls were similar between both pipelines for a majority of drugs assessed; however, the updated pipeline showed a significant increase (two-sample proportion, *P* value < 0.05) in the total number of resistant calls compared to the original pipeline for PZA, BDQ, and CFZ. Substantial agreement (Cohen’s kappa > 0.7) or near-perfect agreement (>0.8) for resistant, susceptible, or no resistance determination was observed for all drugs assessed. And, while the agreement between was high overall, there were some significant differences in marginal homogeneity (*P* value < 0.05) for SM, PZA, BDQ, and CFZ, reflecting the increase in resistance calls by the updated pipeline leading to differences in the distribution of off-diagonal comparisons (Table 2). In the case of MFX and LFX, despite relatively similar overall resistance proportions between the two pipelines, differences in marginal homogeneity were detected reflecting differences in susceptibility calls between the original and updated pipelines.

**Table 2.** Comparison of drug resistance, drug susceptibility, and no resistance determination by pipeline.

Drug	ONT pipeline resistance calls		ONT pipeline concordance	
	Original pipeline n (%)	Updated pipeline n (%)	Cohen’s kappa	Symmetry *P* value
RIF	511 (70.9)	508 (70.5)	0.942	0.411
INH	484 (67.1)	486 (67.4)	0.943	0.083
SM	379 (52.6)	402 (55.8)	0.845	**0.008** ^**A**^
EMB	382 (53.0)	378 (52.4)	0.921	0.133
PZA	267 (37.0)	333 (46.2)^**B**^	0.837	**0.000** ^**A**^
MFX	295 (40.9)	303 (42.0)	0.963	**0.004** ^**A**^
LFX	295 (40.9)	303 (42.0)	0.963	**0.004** ^**A**^
KAN	95 (13.2)	95 (13.2)	0.968	0.754
AMK	56 (7.8)	56 (7.8)	0.933	0.146
CAP	51 (7.1)	53 (7.4)	0.983	0.250
BDQ	4 (0.6)	39 (5.4)^**B**^	0.769	**0.000** ^**A**^
CFZ	0 (0.0)	35 (4.9)^**B**^	0.751	**0.000** ^**A**^
LZD	16 (2.2)	16 (2.2)	0.952	0.813

Drug resistance calls for original and updated pipelines are presented and compared. Paired comparison of resistance, susceptibility, and no resistance determination for original and updated pipeline outputs using Cohen’s Kappa to assess agreement and as exact test of symmetry for all 721 samples assessed.

AMK, amikacin; BDQ, bedaquiline; CAP, capreomycin; CFZ, clofazimine; EMB, ethambutol; INH, isoniazid; KAN, kanamycin; LFX, levofloxacin; LZD, linezolid; MFX, moxifloxacin; ONT, Oxford Nanopore Technology; PZA, pyrazinamide; RIF, rifampicin; SM, streptomycin.

A Significant at *P* < 0.05 for exact test of symmetry.

B Significant at *P* < 0.05 for two-sample test of proportions.

### Diagnostic accuracy comparison

Frequencies for pDST, WGS (using the 2023 WHO mutation catalogue), and composite resistance are presented in Table 3. Prevalence of drug resistance by composite reference ranged from 5% for LZD to 79% for RIF. Performance of the original and updated pipeline for each drug assessed along with their point estimate differences is presented in Table 3. Sensitivities for the original pipeline ranged from 0% for CFZ to 97% for RIF, while for the updated pipeline, sensitivities ranged from 47% for LZD to 96% for RIF. Significant increases in sensitivity (*P* value < 0.05) were observed with the updated pipeline for SM, PZA, BDQ, and CFZ when compared to the original pipeline. Specificities exceeded 90% for all drugs for the original pipeline and 96% for all drugs for the updated pipeline. Both a significant specificity increase for SM and a significant specificity decrease for PZA (*P* value < 0.05) were observed when comparing the updated to the original pipeline. For historical reference, the pDST, WGS using the 2021 WHO mutation catalogue, and corresponding composite resistance, along with associated performance metrics are presented in Supplementary Data Table S1.

**Table 3.** Prevalence of drug-specific resistance as determined by pDST, WGS, and composite reference for all 721 samples evaluated.

Drug	Reference drug resistance			Original ONT pipeline							Updated ONT pipeline							Point estimate difference	
	pDST n (%)	WGS n (%)	Composite n (%)	n	TP	TN	FN	FP	Sensitivity (95% CI)	Specificity (95% CI)	n	TP	TN	FN	FP	Sensitivity (95% CI)	Specificity (95% CI)	Sensitivity	Specificity
RIF	533 (73.9)	561 (77.8)	572 (79.3)	649	511	120	18	0	96.6 (94.7, 97.8)	100.0 (96.9, 100.0)	651	506	119	24	2	95.5 (93.4, 96.9)	98.3 (94.2, 99.5)	−1.1	−1.7
INH	537 (74.5)	513 (71.2)	538 (74.6)	649	483	142	23	1	95.5 (93.3, 97.0)	99.3 (96.1, 99.9)	651	483	141	24	3	95.3 (93.1, 96.8)	97.9 (94.1, 99.3)	−0.2	−1.4
SM	473 (65.6)	447 (62.0)	490 (68.0)	643	362	170	94	17	79.4 (75.4, 82.8)	90.9 (85.9, 94.2)	643	395	180	61	7	86.6 (83.2, 89.4)	96.3 (92.5, 98.2)	7.2*	5.3*
EMB	401 (55.6)	397 (55.1)	462 (64.2)	669	379	222	65	3	85.4 (81.8, 88.3)	98.7 (96.2, 99.5)	664	375	219	67	3	84.8 (81.2, 87.9)	98.6 (96.1, 99.5)	−0.5	0
PZA	380 (52.7)	344 (47.7)	394 (54.7)	600	267	245	88	0	75.2 (70.5, 79.4)	100.0 (98.5, 100.0)	605	327	241	31	6	91.3 (88.0, 93.8)	97.6 (94.8, 98.9)	16.1*	−2.4*
MFX	313 (43.4)	315 (43.7)	325 (45.1)	669	293	354	20	2	93.6 (90.3, 95.8)	99.4 (98.0, 99.8)	665	300	347	15	3	95.2 (92.3, 97.1)	99.1 (97.5, 99.7)	1.6	−0.3
LFX	314 (43.6)	315 (43.7)	327 (45.4)	668	293	351	22	2	93.0 (89.7, 95.3)	99.4 (98.0, 99.8)	664	300	344	17	3	94.6 (91.6, 96.6)	99.1 (97.5, 99.7)	1.6	−0.3
KAN	104 (14.4)	106 (14.7)	128 (17.8)	617	94	503	19	1	83.2 (75.2, 89.0)	99.8 (98.9, 100.0)	615	94	502	18	1	83.9 (76.0, 89.6)	99.8 (98.9, 100.0)	0.7	0
AMK	60 (8.3)	57 (7.9)	63 (8.7)	680	54	617	7	2	88.5 (78.2, 94.3)	99.7 (98.8, 99.9)	674	54	611	7	2	88.5 (78.2, 94.3)	99.7 (98.8, 99.9)	0	0
CAP	60 (8.3)	54 (7.5)	67 (9.3)	639	49	576	12	2	80.3 (68.7, 88.4)	99.7 (98.7, 99.9)	637	51	574	10	2	83.6 (72.4, 90.8)	99.7 (98.7, 99.9)	3.3	0
BDQ	42 (5.8)	46 (6.4)	57 (7.9)	638	4	591	43	0	8.5 (3.4, 19.9)	100.0 (99.4, 100.0)	640	37	588	13	2	74.0 (60.4, 84.1)	99.7 (98.8, 99.9)	65.5*	−0.3
CFZ	40 (5.6)	45 (6.3)	54 (7.5)	638	0	593	45	0	0.0 (0.0, 7.9)	100.0 (99.4, 100.0)	643	32	593	15	3	68.1 (53.8, 79.6)	99.5 (98.5, 99.8)	68.1*	−0.5
LZD	33 (4.6)	15 (2.1)	34 (4.7)	669	16	637	16	0	50.0 (33.6, 66.4)	100.0 (99.4, 100.0)	667	15	634	17	1	46.9 (30.9, 63.6)	99.8 (99.1, 100.0)	−3.1	−0.2

Diagnostic accuracy of original and updated pipelines including the total number of samples assessed with resistant or and susceptible calls for both original and updated pipelines compared to the composite reference, and corresponding true positive, true negative, false negative, false positive, drug-specific sensitivity and specificity, and finally sensitivity and specificity point estimate differences.

* *P* value < 0.05.

AMK, amikacin; BDQ, bedaquiline; CAP, capreomycin; CFZ, clofazimine; EMB, ethambutol; FN, false negative; FP, false positive; INH, isoniazid; KAN, kanamycin; LFX, levofloxacin; LZD, linezolid; MFX, moxifloxacin; ONT, Oxford Nanopore Technology; pDST, phenotypic drug susceptibility testing; PZA, pyrazinamide; RIF, rifampicin; SM, streptomycin; TN, true negative; TP, true positive; WGS, whole genome sequencing.

### Diagnostic yield

To evaluate resistance detection yield, the number of composite resistant samples identified as resistant (true positive) by each pipeline out of all samples assessed was compared for each drug (Table 4). Significant increases in resistance detection yield were identified for SM (6.7%), PZA (15.2%), BDQ (57.9%), and CFZ (59.3%) when comparing the updated pipeline to the original pipeline.

**Table 4.** Resistance detection yield.

Drug	Composite R (n)	Original ONT pipeline true positive, n (%)	Updated ONT pipeline true positive, n (%)	Difference (%)
RIF	572	511 (89.3)	506 (88.5)	−0.9
INH	538	483 (89.8)	483 (89.8)	0.0
SM	490	362 (73.9)	395 (80.6)	6.7*
EMB	463	379 (81.9)	375 (81.0)	−0.9
PZA	394	267 (67.8)	327 (83.0)	15.2*
MFX	325	293 (90.2)	300 (92.3)	2.2
LFX	327	293 (89.6)	300 (92.6)	3.0
KAN	128	94 (73.4)	94 (73.4)	0.0
AMK	63	54 (85.7)	54 (85.7)	0.0
CAP	67	49 (73.1)	51 (76.1)	3.0
BDQ	57	4 (7.0)	37 (64.9)	57.9*
CFZ	54	0 (0.0)	32 (59.3)	59.3*
LZD	34	16 (47.1)	15 (44.1)	−2.9

* *P* value < 0.05.

AMK, amikacin; BDQ, bedaquiline; CAP, capreomycin; CFZ, clofazimine; EMB, ethambutol; INH, isoniazid; KAN, kanamycin; LFX, levofloxacin; LZD, linezolid; MFX, moxifloxacin; ONT, Oxford Nanopore Technology; PZA, pyrazinamide; R, resistance detected; RIF, rifampicin; SM, streptomycin

## DISCUSSION

tNGS provides a sensitive and cost-effective method for detecting DR-TB directly from clinical specimens with faster turnaround times compared to pDST and WGS from culture.^15,16^ The ability to perform bioinformatic updates as new resistance-associated mutations are identified within gene targets already included in the assay enables tNGS solutions to evolve in real time.^17^ Due to the paired design of this study, we were able to directly compare sequencing success, resistant call agreement, diagnostic accuracy, and diagnostic yield between the original and updated pipelines for the ONT TB Drug Resistance Test. Our study demonstrated that updates to only the bioinformatic pipeline resulted in significant increases in both the sensitivity and yield of resistance detection for SM, PZA, BDQ, and CFZ when compared to the original pipeline.

### Sequencing success

The ability to interpret sequencing information is driven by multiple factors within the bioinformatics pipeline including coverage thresholds, quality of variant call, and preset quality thresholds. The comparison of sequencing success – defined as the ability of the pipeline to produce interpretable results that can be identified as resistant or susceptible from sequence data – between the original and updated pipelines indicated no significant overall improvement. These results were expected as identical FastQ files were used for both pipelines.

### Resistance call agreement

Pairwise comparison of resistance calls indicated that there was substantial (>0.7) or near-perfect (>0.8) agreement in resistant, susceptible, and no resistance determination calls between the original and updated pipelines for all drugs assessed. There were, however, significant differences in the off-diagonal calls for SM, PZA, MFX, LFX, BDQ, and CFZ. For SM, PZA, BDQ, and CFZ, these marginal differences were driven exclusively by improvements to sensitivity; that is, specimens identified as susceptible by the original pipeline were identified as resistant by the updated pipeline. Differences in marginal homogeneity for MFX and LFX were attributable to susceptibility calls by the original pipeline being called as either resistant or no resistance determination by the updated pipeline.

### Diagnostic accuracy comparison

Diagnostic accuracy, measured by sensitivity and specificity relative to a composite reference standard, was comparable between the original and updated pipelines for RIF, INH, EMB, MFX, LFX, KAN, AMK, CAP, and LZD. Significant increases in sensitivity were observed for SM (+7.2%) and PZA (+16.1%), along with an increase in specificity for SM (+5.4%). While PZA specificity decreased slightly (−2.4%), the decrease was marginal compared to the gain in PZA sensitivity. Dramatic gains were achieved in sensitivity for BDQ (+65.5%) and CFZ (+68.1%), highlighting the enhanced performance of the updated pipeline for these critical drugs. While the specific details of the pipeline updates are proprietary, the gains in sensitivity were likely driven by the addition of variants being interpreted as resistant in the updated pipeline, as well as the addition of ‘interpretation rules’ such as counting loss of function mutations as associated with resistance in the updated pipeline.^12^ This underscores the unique and real-time impact tNGS pipeline updates can have on improving detection of DR-TB. Sensitivity and specificity results from the updated pipeline were similar for INH, SM, PZA, KAN, AMK, and CAP compared to point estimates presented in a meta-analysis by Schwab et al.^17^ for multiple tNGS solutions. However, the updated pipeline MFX sensitivity estimate (95.2% [95% CI: 92.3–97.1]) was markedly higher compared to the meta-analysis sensitivity estimate of 87.6% (95% CI: 74.0–94.9). Interestingly, the updated pipeline EMB sensitivity estimate was significantly lower (85.0% [95% CI: 81.4–88.1]) compared to the meta-analysis EMB sensitivity (96.2% [95% CI: 92.3–98.6]), and in contrast the updated pipeline EMB specificity was significantly higher (98.6% [95% CI: 96.1–99.5]) than the meta-analysis EMB specificity (93.1% [95% CI: 88.0–96.3]).^17^

### Diagnostic yield

Diagnostic yield for detection of DR-TB is a key metric for evaluating the performance of novel diagnostics and assessing their potential for improved treatment outcomes.^18,19^ For both SM and PZA, there was a significant increase in yield for resistance detection, 6.7% and 15.2%, respectively, among study participants when comparing true positives detected by the updated pipeline to the original pipeline. For BDQ and CFZ, there was a dramatic increase in yield for resistance detection, 57.9% and 59.3%, respectively. These increases in resistance detection demonstrate the unique and real-time impact tNGS pipeline updates can have on diagnostic yield.

Our study has some limitations. Due to the proprietary nature of updates to the bioinformatic ONT pipeline, internal modifications to the pipeline could not be assessed and therefore we were unable to explain the specific cause when changes in end-user results occurred. Updates to the pipeline were implemented primarily to improve sensitivity to BDQ, CLF, and PZA resistance.^14^ Thus, the analyses described in this study were not designed to calculate specific mutation sensitivities nor determine mutation-based discordance but instead focused on differences in drug resistance interpretation to compare the original and updated pipelines. A general limitation to evaluating pipeline updates that include modifications to thresholds for control validation or variant classifications is that end-user differences may not be reproducible across datasets without specific knowledge of modifications. However, by comparing end-user results using the same dataset we were able to evaluate differences attributable only to pipeline updates. An additional limitation to comparing end-user result changes when only pipeline modifications have been implemented is that performance improvements are restricted to new variants and interpretation rules based on variants that are discoverable within the gene targets included in the assay.

## CONCLUSION

This study demonstrates that substantial diagnostic improvements are achievable through bioinformatics pipeline updates. While no significant differences in overall sequencing success between the original and updated pipelines were observed, the updated pipeline showed marked enhancements in sensitivity, for BDQ and CFZ, and significant increases in diagnostic yield for SM, PZA, BDQ, and CFZ. These improvements highlight the potential of tNGS bioinformatics to be rapidly adapted to accommodate emerging resistance mutations within included gene targets, offering a scalable and dynamic solution for DR-TB diagnostics. This adaptability could fundamentally transform diagnostic approaches, ensuring alignment with evolving resistance landscapes and optimising clinical decision-making in real time. This work also highlights the need for ongoing revisions of analysis pipelines to maximise test sensitivity, informed by the expanding catalogue of resistance-associated mutations.

## Supplementary Material


