# Supplementary material for: Comparing end-user diagnostic outputs from a commercial tNGS pipeline for Mycobacterium tuberculosis drug resistance detection
Source: IJTLD Open. 2025 Nov 12;2(11):677–84. doi: 10.5588/ijtldopen.25.0245 (PMC12617087; doi:10.5588/ijtldopen.25.0245)
Supplement: Supplementary file 1 [file ijtldopen25-0245_supplementarydata1.pdf]

SUPPLEMENT

Table S1. Prevalence of drug specific resistance as determined by pDST, WGS catalogue v1 (2021), and composite reference using WGS catalogue version 1 (2021) for 721 samples evaluated. Performance metrics of original pipeline compared to composite reference using WGS version 1 (2021).

	Reference drug resistance			ONT original compared to Composite w/ WGS v1						
Drug	pDST n (%)	WGS v1 n (%)	Composite w/ WGS v1 n (%)	n	TP	TN	FN	FP	Sensitivity (95%CI)	Specificity (95%CI)
**RIF**	533 (73.9)	567 (78.6)	578 (80.2)	649	511	115	23	0	95.7 (93.6, 97.1)	100.0 (96.8, 100.0)
**INH**	537 (74.5)	512 (71.0)	538 (74.6)	649	483	142	23	1	95.5 (93.3, 97.0)	99.3 (96.1, 99.9)
**SM**	473 (65.6)	457 (63.4)	493 (68.4)	643	361	166	98	18	78.6 (74.7, 82.2)	90.2 (85.1, 93.7)
**EMB**	401 (55.6)	413 (57.3)	464 (64.4)	669	380	222	65	2	85.4 (81.8, 88.4)	99.1 (96.8, 99.8)
**PZA**	380 (52.7)	367 (50.1)	400 (55.5)	600	267	242	91	0	74.6 (69.8, 78.8)	100.0 (98.4, 100.0)
**MFX**	313 (43.4)	310 (43.0)	321 (44.5)	669	293	358	16	2	94.8 (91.8, 96.8)	99.4 (98.0, 99.8)
**LFX**	314 (43.6)	310 (43.0)	324 (44.9)	668	293	354	19	2	93.9 (90.7, 96.1)	99.4 (98.0, 99.8)
**KAN**	104 (14.4)	106 (14.7)	128 (17.8)	617	94	503	19	1	83.2 (75.2, 89.0)	99.8 (98.9, 100.0)
**AMK**	60 (8.3)	57 (7.9)	63 (8.8)	680	54	617	7	2	88.5 (78.2, 94.3)	99.7 (98.8, 99.9)
**CAP**	60 (8.3)	54 (7.5)	67 (9.3)	639	49	576	12	2	80.3 (68.7, 88.4)	99.7 (98.7, 99.9)
**BDQ**	42 (5.8)	n/a	42 (5.8)*	638	2	602	32	2	5.9 (1.6, 19.1)	99.7 (98.8, 99.9)
**CFZ**	40 (5.6)	n/a	40 (5.6)*	638	0	606	32	0	0.0 (0.0, 10.7)	100.0 (99.4, 100.0)
**LZD**	33 (4.6)	14 (1.9)	34 (4.7)	669	16	637	16	0	50.0 (33.6, 66.4)	100.0 (99.4, 100.0)

*pDST only
